# Supplementary material for: Are carbon emissions trading and green financial instruments synergistic? -Comprehensive quantitative research based on content analysis
Source: PLoS One. 2024 Mar 7;19(3):e0298601. doi: 10.1371/journal.pone.0298601 (PMC10919601; doi:10.1371/journal.pone.0298601)
Supplement: S1 Data — (ZIP) [file pone.0298601.s001.zip › Data/地级市Z_F工作报告.zip!/┴╔─■/╠·┴δ╩╨/└┤╫╘╣1⁄2╓┌║┼í╛┬φ┐╦╩2╛▌═°í┐_2010.docx]

各位代表：

现在，我代表市人民政府向大会报告工作，请予审议，并请市政协委员和其他列席会议的同志提出意见。

一、2009年工作回顾

过去的一年，是铁岭经济社会发展实现新突破、再创新辉煌的一年。我们在省委、省政府和市委的正确领导下，在市人大依法监督、市政协民主监督与大力支持下，团结和带领全市各族人民，全面贯彻党的十七大和十七届三中、四中全会精神，坚持以邓小平理论和“三个代表”重要思想为指导，以科学发展观为统领，积极应对国际金融危机的严峻挑战，全力做好保增长、保民生、保稳定各项工作，经济社会发展取得显著成就，全面完成了市六届人大一次会议确定的各项任务，做到了化危为机，实现了“弯道超车”。

—— 综合经济实力再上新台阶。预计全市地区生产总值实现653亿元，增长18%，人均GDP突破3000美元。地方财政一般预算收入48亿元，增长45%。规模以上工业增加值实现325亿元，增长25%。全社会固定资产投资完成780亿元，增长54%。社会消费品零售总额实现195亿元，增长20%。金融机构存款余额572亿元，贷款余额424.5亿元，分别增长26.6%和24.8%。城镇居民人均可支配收入12960元，增长18.8%；农民人均纯收入7000元，增长15%。万元GDP综合能耗、水耗分别下降5%和4%，二氧化硫、化学需氧量排放量分别下降7.3%和3.5%。主要经济指标在全省位次明显前移，地区生产总值、地方财政一般预算收入、规模以上工业增加值分别提升到第9位、第8位和第7位。

—— 对外开放实现历史性突破。引进域外投资项目993个，到位资金530亿元。煤矸石发电、合力专用车、中利电缆等一批项目建成投产，北祥重工、冀东专用车、哈尔滨深冷等一批项目开工建设。实际利用外资1.7亿美元，增长43%。外贸出口4.2亿美元，增长176%。积极抢抓国家扩内需、保增长的机遇，争取各类资金65.6亿元。特别令人振奋的是，香港恒基兆业、中航工业集团等世界500强企业落户我市，铁岭开启了与巨人同行的新时代。

—— 工业经济步入优化发展新阶段。初步实现了工业集中向产业集聚、传统工业向新型工业化跨越。产业集群迅速崛起。专用车生产基地创造了奇迹，开工建设项目51个，在全国产生重大影响。换热设备产业基地一炮打响，32个项目建成投产。起重机械产业集群进一步做大，生产企业发展到118家。有色金属、橡塑制品等产业集群日益壮大。新兴产业高起点起步。35万千瓦风力发电项目并网发电，年产5万辆电动车项目开工建设。传统产业改造升级步伐加快。实施技术改造项目550项，完成投资210亿元。规模以上工业企业发展到1459户，销售收入亿元以上企业达到200户、10亿元以上企业达到25户。主导产业更加突出，先进制造业占工业经济的比重提升到32%。科技含量明显提高。实现高新技术产品产值161亿元，增长50%，涌现出三洋重工等一批国内外领先企业。园区功能进一步完善。投入园区基础设施建设资金25亿元，园区面积增加到96平方公里，专业分工更加明确，配套设施更加齐备，园区环境更加优美，承载能力进一步增强。

—— 城市面貌展现全新形象。凡河新城区建设再掀新高潮。沈铁城际铁路开工建设，新城区客运站主体封顶，新调公路、东北物流城至新城区连接线建成通车。22平方公里区域基础设施基本建成，建筑总面积达到361万平方米。莲花湖国家城市湿地公园获建设部批准，莲花湖湿地恢复工程荣获中国人居环境范例奖。地热勘查取得成功。市、县行政中心顺利南迁，掀开了铁岭历史崭新一页。浅水湾1号分期交付使用，水木华园一期盛大开盘。以一卡通为标志的数字城市建设正式启动。新城区对外产生了强烈的吸引力，恒基兆业、上海弘久、中国新城市等知名房地产企业纷纷落户。银州区城市品位明显提升。11件实事全部完成，成功跻身国家级园林城市行列，被评为全国和谐社区建设示范城区。副中心城市建设成效显著。开原市进入国家级园林城市，城市建设经验在全省推广。调兵山市城市功能进一步完善，被评为全国平安建设先进市。昌图县县城改造取得突破性进展，城市面貌焕然一新。西丰县、清河区城市建设也取得了新成效。以新台子、腰堡、庆云堡、八面城等为代表的新市镇建设步伐加快。辽河生态治理和节能减排取得阶段性成果，水质明显改善，空气优良天数在全省名列前茅。

—— 农业农村经济全面发展。在百年不遇的旱灾之年仍然做到了减产不减收。农业经济结构调整力度进一步加大。四个100万亩工程扎实推进，保护地蔬菜、优质水稻、花生、榛子种植面积分别达到95万亩、95万亩、85万亩和99万亩。畜牧业发展实现重大突破，实现产值158亿元，增长24.4%。农业产业化迈出新步伐。新建固定资产投资2000万元以上项目29个，规模以上龙头企业发展到196家，农业产业化率达到52%。农民的组织化程度显著提高。农民专业合作社发展到1277个，合作社成员10万人，有组织地转移输出劳动力5万人。农业科技和农产品质量安全工作取得新进展。推广农业新品种、新技术200项，铁岭大米、铁岭榛子、铁岭花生正式注册为国家地理标志性商标，铁岭县、开原市被评为全国科技进步先进县。农业基础设施得到加强。建设骨干工程365项，完成农建土石方5153万立方米，对38座小型水库进行了除险加固，有望连续5年夺得省大禹杯。综合机械化水平达到53.6%。农业政策性保险工作走在全省前列。县域经济发展迅猛。开原市“三化”进程实现重大突破，有望进入全国百强县。调兵山市城乡一体化成效显著，铁岭县成为名副其实的工业大县，昌图县工业经济长足进步，西丰县特色产业发展壮大。承诺为农民办的14件实事全部完成。

—— 现代服务业呈现勃勃生机。铁岭新城现代服务业基地建设取得重大进展。东北物流城一期工程竣工，首批商家入驻，机动车交易市场等二期工程开工建设。成功承办全国首届优秀农产品流通经纪人表彰大会，东北物流城被中华全国供销总社确定为中国农产品流通经纪人创业基地和中国优质农产品东北展示展销中心。北方金融后台服务基地建设取得实质性进展。职业教育基地初具规模，在校生达到1.5万人，招生、就业形势喜人。沈铁工业走廊服务产业带蓬勃发展。银州区商贸中心地位凸显，兴隆百货、大商新玛特、乐购等购销两旺，五星级粤海国际酒店即将开业，城区经济呈现良好发展势头。开原五金城、建材城、汽配城等专业市场形成规模，西丰鹿产品进出口加工贸易保税试点区获得批准，家和美商城落户昌图，调兵山兀术花园小区投入使用。万村千乡市场工程、农村新网工程和家电下乡活动扎实推进，农村消费市场更趋活跃。粮食清仓查库工作成效显著，建设新型农户科技储粮仓2万套，农民卖粮难问题得到有效解决。房地产业空前高涨。新开工面积859万平方米，实现收入162亿元。旅游业快速发展。共接待境内外游客874万人次，实现收入60亿元。

—— 体制机制创新迈出新步伐。融资工作实现新突破。成功发行15亿元企业债券，财京公司上市重组进展顺利。借、用、管、还一体化投融资体系基本建立。土地招拍挂力度加大，市本级出让土地323万平方米，出让价款20亿元。金融服务体系建设成效显著，金融生态环境明显改善。国有商业银行支持地方经济发展的作用日益增强，市商业银行成功增资扩股。铁岭新星村镇银行运行良好，昌图、开原村镇银行正式开业，村镇银行数量居全省之首。担保机构发展到16家，组建小额贷款公司12家。部门预算、国库集中收付、政府采购等制度不断完善。行政管理体制改革取得重大进展。政府信息公开工作全面推进，行政权力公开透明运行取得良好成效，公共行政服务四级联动新机制开始启动，行政事业性收费实现目录制管理，政府机构改革积极稳妥推进，事业单位改革进一步深化。新城区管理体制趋于完善，政府主导、市场运作、社会参与模式已经确立，机关后勤服务和物业管理实现社会化，做到了老体制不进新城区。

—— 民生工程成效显著。筹集资金2.7亿元，开展实名制就业培训29365人，安置实名制就业76981人、大学生就业4024人。筹集资金15.4亿元，为全市133590名企业离退休人员按时发放了养老金。西丰县新型农村养老保险开始实施。筹集资金近3亿元，将79760名困难企业退休人员纳入医疗保险范围。筹集资金2700万元，为城乡困难群众实施了医疗救助。筹集资金2.4亿元,确保了71956名城市低保对象、89178名农村低保对象低保金按时足额发放。筹集资金2943万元，使13623名农村五保供养对象做到了应保尽保。筹集资金2587万元，改造农村低保户D级危房2276户，至此，全市农村低保户D级危房全部改造完毕。筹集资金9亿元，建设经济适用房48万平方米、廉租房15万平方米，解决了12578户困难居民的住房问题。筹集资金944万元，新建、改扩建中心敬老院6所。筹集资金957万元，对5519户城乡低保边缘户实施了救助。筹集资金2058万元，对城乡低保对象实施了临时救济。筹集资金2370万元，保障了受灾群众的基本生活。筹集资金2848万元，建设农村饮水安全工程256处，解决了24万人饮水安全问题。筹集资金200万元，新建、改扩建农村社区服务中心60个。

—— 社会事业全面繁荣。教育年活动硕果累累。银冈学院、铁岭县高中顺利南迁，卫生学校、技师学院晋升高职通过省评估组验收。铁岭高中与全国50余所重点大学建立了自主招生合作关系。建设农村九年一贯制学校22所，改造学校50所、4.8万平方米。我市荣获首届全国教育改革创新特别奖。医药卫生体制改革正式启动，公共卫生服务体系建设得到加强，甲型H1N1流感防控工作成效显著，新型农村合作医疗制度不断巩固。公共文化体系进一步完善，群众性文化活动丰富多彩，工笔画在全国展览中连连获奖。全民健身活动蓬勃开展，竞技体育再创佳绩。广播电视宣传质量和舆论导向作用进一步提升，农村有线广播电视数字化暨文化信息资源共享工程建设经验在全国推广。人口和计划生育工作得到加强，出生人口素质进一步提高。

—— 社会保持和谐稳定。平安铁岭建设成绩斐然。打黑除恶专项斗争取得重大成果，社会治安防控体系更加健全，成为全省最稳定地区之一，出色完成国庆安保任务。深入开展安全生产年活动，安全生产形势总体平稳。搭建信访接待中心平台，深入开展领导干部大接访活动，一批信访积案得到化解。食品安全监管全面加强，药品监管实现信息化。应急管理工作体系日益完善，应急保障能力不断增强。政府法制建设得到加强，依法行政水平进一步提高。民族关系和谐融洽，宗教活动依法有序开展。人大代表建议、政协提案办理质量明显提高，双安水厂和柴河水库水源保护议案办理工作得到市人大代表好评。

外事、侨务、台办、统计、审计、物价、综合执法、档案、气象、国税、地税、电业、规划、出入境检验检疫、烟草、人防、国家安全、武警、消防、新闻出版、地震、政务接待、残联、科协、商业保险、邮政、通信、驻外联络、市志、老龄、仲裁等工作也都取得了新成绩。

各项成就的取得，是省委、省政府和市委正确领导的结果，是全市各族人民智慧和汗水的结晶。在此，我代表市人民政府，向辛勤工作在各条战线上的全市各族人民，向积极支持政府工作的市人大代表、市政协委员、各民主党派、工商联、无党派人士、人民团体、中省直驻铁单位及社会各界人士，向解放军驻铁部队指战员和武警官兵，向所有关心、支持、参与新铁岭大铁岭建设的广大客商和海内外朋友表示衷心的感谢并致以崇高的敬意！

在看到成绩的同时，也要清醒地看到存在的主要矛盾和问题。一是经济发展方式还比较粗放，经济结构不尽合理，工业自主创新能力不强，节能减排和环境保护压力较大。二是由于区位条件、产业基础等原因，区域经济发展不平衡，差距进一步拉大。三是人民群众收入水平不高，全市城乡还有相当数量的困难群体。四是政府职能转变还不适应新形势、新任务的要求，软环境还有不尽如人意之处。这些问题必须引起我们的高度重视，并在今后的工作中采取有力措施认真加以解决。

二、2010年的主要工作

今年是实施“十一五”规划的最后一年，也是谋划“十二五”发展、实现铁岭全面振兴的关键之年，做好各项工作意义重大。当前，我们正面临着错综复杂、利弊交织的发展环境。一方面，国际金融危机的影响依然存在，我国经济回升的基础还不稳固，经济社会发展的不确定因素还很多。另一方面，中央继续实施积极的财政政策和适度宽松的货币政策，省委、省政府深入实施建设沈阳经济区和突破辽西北战略，都将给我市带来新的发展机遇。特别是近几年来我市拓宽发展空间，培育发展载体，创优发展环境，形成了更好更快的发展基础，增强了抗御风险的能力。只要我们认清形势，趋利避害，抢抓机遇，乘势而上，铁岭完全能够在今年乃至今后一个时期保持强劲的发展势头，在激烈的竞争中继续保持领先位置。今年政府工作的指导思想是：全面贯彻党的十七大和十七届三中、四中全会及中央经济工作会议精神，以邓小平理论和“三个代表”重要思想为指导，以科学发展观为统领，以提高经济增长质量和效益为目标，以转变经济发展方式和调整经济结构为主线，以推进改革开放和自主创新为动力，以注重改善民生、保持社会和谐稳定为保障，以推进工业化、城镇化为重点，努力实现科学发展、创新发展、和谐发展的新突破，为“十二五”高起点开局、实现“四个跨越”奠定坚实基础。

主要预期目标是：地区生产总值800亿元，增长18%。地方财政一般预算收入60亿元，增长25%。全社会固定资产投资1000亿元，增长35%。规模以上工业增加值395亿元，增长20%。社会消费品零售总额238亿元，增长22%。城镇居民人均可支配收入15300元，增长18%；农民人均纯收入7910元，增长13%。城镇登记失业率控制在3.8%以下，人口自然增长率控制在3.3‰以内。万元GDP综合能耗下降3%，水耗下降2%，二氧化硫和化学需氧量排放量分别减少3.9%和0.5%。

为实现上述目标，突出抓好以下10项重点工作：

（一）深入开展项目年活动，全方位扩大对外开放

继续加大招商引资力度。重点围绕沈铁工业走廊、凡河新城区等发展载体，强化产业集群招商、主题概念招商、新兴产业招商和飞地招商，优化对外开放结构。组织好境内外系列招商活动，确保全年引进亿元以上项目100个，开工建设项目500个，投产达效项目300个，引进内资650亿元，实际利用外资3亿美元。对事关全局的100个重大项目，实施领导包扶责任制，务求取得突破性进展。加快海关申办进程，努力扩大出口，确保外贸出口实现5亿美元。

（二）大力调整工业结构，加速推进产业升级

加快调整产业结构。集中全市资源建设汽车城，专用车生产基地确保100个项目开工建设，50个项目建成投产，完成投资300亿元，形成1000亿元的生产能力。换热设备产业基地引进项目150个，80个项目建成投产，完成投资70亿元。起重机械生产基地开工建设项目80个，完成投资80亿元。同时，积极培育农产品加工、矿山设备、有色金属、橡塑制品、精密铸造、阀门等产业集群。发展产业集群，要突出新兴产业。新兴产业是新的经济增长点，代表着希望和未来。要发挥现有优势，集中力量在先进制造、新能源、新材料、生物化工、环保产业、服务外包6个重点领域取得突破，将高新区打造成全市新兴产业示范区，将开发区打造成全市新型工业化道路的先导区。加快发展低碳经济和循环经济。加快调整企业结构。继续开展科技创新年活动，深入实施“1123工程”，重点抓好嘉旭铜业年产10万吨电解铜等100个重大技改项目，完成投资200亿元，实现高新技术产品产值240亿元，增长50%以上。制定出台优惠政策，鼓励企业增资扩股、兼并重组、做大做强，新增销售收入亿元以上企业50户、10亿元以上企业10户。扶持企业自主创新，开发新产品330项，新创省级名牌产品6个、国家级名牌产品1个。进一步完善园区功能，加强园区服务体系建设。

（三）积极推进沈铁同城化，加快提升城镇化水平

把城镇化作为应对国际金融危机、扩大国内需求、调整经济结构的重要载体，为经济社会长期又好又快发展开拓新空间、增添新活力。以“一区两带”建设为重点，加快沈铁同城化步伐。一区即包括凡河新城区和银州区在内的中心城区，将其建成100万人口的大城市，打造成独具魅力的中国北方水城和沈阳经济区休闲度假中心。依托中心城区发展两带，即以高新区、开发区、专用车生产基地、东北物流城、懿路、腰堡、凡河、银州工业园区为支撑的沈铁产业带；以新台子、腰堡、凡河三个新市镇为结点的沈铁城镇带。积极探索工业化与城镇化有机结合的发展模式，使工业化与城镇化相互促进、协调发展，努力实现产业振兴与城市繁荣，率先实现沈铁同城化。全力推进凡河新城区建设，在人口聚集、产业聚集、生产要素聚集上实现新突破。突出抓好恒基兆业天水河商业步行街和莲花湖高档住宅、上海弘久浅水湾2号等20个新建项目和医学专科学校等25个续建项目，完成投资100亿元。加快新城区超市、酒店、文化、体育、旅游等服务设施建设，完善城市功能，提升城市品位。集中力量抓好沈铁城际铁路、辽宁中环新城区至新民高速公路、沈铁二号公路等交通走廊建设。进一步加大银州区改造力度，重点办好10件实事。1.为3100户城市困难居民解决廉租房。2.改造城市东南出口、西南出口，新建玉深南路，改造长青路东段。3.对柴河大桥实施加固。4.建设汇工街、北环路等5处带状游园。5.修建恢复龙首山生态通道，实施街路节点绿化和立体绿化。6.修复秀峰塔。7.实施610厂给水设施改造和30栋弃管楼供水管线改造。8.完善污水收集系统，建设1万立方米调峰蓄水池。9.改造小街小巷。10.继续实施铁西一铁道门等棚户区改造。理顺中心城区管理体制，向城区下放权力，促进城区经济快速发展。加大环境综合整治力度，全力争创国家环保模范城和国家卫生城。加快推进以物联网为切入点的数字城市建设，提高城市现代化管理水平。推广银州区和谐社区建设经验，全面提高社区建设水平。加快副中心城市建设。启动开原市滨河新区建设，早日将开原建成50万人口的中等城市，使凡河新城区与开原滨河新区双峰并峙、比翼齐飞。继续提升调兵山市、清河区城市功能，争取昌图、西丰进入省级园林城市。加快20个中心集镇基础设施建设，提高综合承载能力。取消城乡户口限制，促进农业转移人口在城镇就业和落户。进一步加强辽河生态治理，加大重点污染源环境监管力度，全面完成“十一五”节能减排任务。

（四）加快农业产业化步伐，优化农业经济结构

加大农业经济结构调整力度。按照一县一业的布局，着力培育畜牧、蔬菜、榛子三大主导产业，加快建设铁岭县都市观光农业、开原市开西现代农业、昌图县八面城现代畜牧业、西丰县鹿产业四大示范区。继续推进百亿斤粮食工程和四个100万亩工程。按照建设全省畜牧产业第一市的目标，加快推进生猪、肉牛、肉鸡三大主导产业开发，实现畜牧业产值200亿元。深化林权制度改革，大力发展榛子产业，实现林业产值65亿元。加快推进农业产业化进程。新上固定资产投资2000万元以上项目60个，实现农业产业化产值375亿元。进一步提高农民的组织化程度，新建60个标准示范农民专业合作社，实现龙头企业与合作社的对接联动。加强农业基础设施建设，完成土石方2500万立方米，建设骨干工程160项，完成人工造林38.5万亩。进一步提高农业机械化水平。加快发展县域经济。全面推广开原市推进工业化、城镇化、农业现代化的经验，在全市形成一个学习开原、赶超开原、竞相发展的新局面，确保开原市进入全国百强县，调兵山市、铁岭县全力向全国百强县冲刺，昌图县实现位次前移。继续举全市之力支持西丰县，尽快实现经济社会发展的新突破。

（五）大力发展现代服务业，努力提高服务业在国民经济中的比重

加快铁岭新城现代服务业基地建设。创造条件，放宽政策，全力支持东北物流城建设，重点抓好建材大世界、机动车交易市场、化肥交易中心、粮食和农副产品交易中心等二期工程。积极推进北方金融后台服务基地建设。扩大职业教育基地规模，在校生达到5万人以上。加快沈铁工业走廊服务产业带建设。巩固银州区商贸中心地位，进一步提升服务业品位。完善开原市五金城等专业市场，逐步建成区域性物流集散地。抓好调兵山市五星级酒店建设，推动服务业提档升级。充分发挥西丰县土特产品交易中心作用，叫响“中国鹿乡”品牌。加大昌图滨河商业金廊开发力度，繁荣昌图城区经济。加强清河区服务业基础设施建设。加快发展旅游业。抓好重点旅游项目建设，全年接待境内外游客力争突破1000万人次，实现旅游业总收入78亿元。继续推进万村千乡市场工程和农村新网工程，组织好家电、汽车、摩托车下乡活动，大力促进城乡消费。建设新型农户科技储粮仓2万套，积极做好粮食收购工作。繁荣活跃房地产业，开发房地产1000万平方米，实现收入212亿元。服务业增加值实现240亿元，增长26%。

（六）切实加强财税金融工作，为经济社会发展提供强力支撑

狠抓增收节支。大力组织财政收入，做到应收尽收。加强非税收入征管，增强政府统筹能力。加大社保基金征缴力度，做到足额征收、及时入库。牢固树立过紧日子思想，严格控制一般性支出，切实降低行政成本。优化财政支出结构，完善公共财政体系，加大向民生倾斜力度。继续拓宽融资渠道。加大村镇银行组建力度，力争每个县（市）都有一家村镇银行。新组建小额贷款公司10家。鼓励金融机构在重点工业园区增设经营网点，加大对中小企业、新兴产业的支持力度。积极推进担保机构增资扩股，鼓励兴办民营担保机构。继续加大资产整合力度，按照“资源资产化、资产资本化、资本证券化”的要求，做大融资平台，努力经营城市。加强投融资监管，进一步完善借、用、管、还管理体系。提高企业直接融资能力，力争1—2家企业上市。

（七）更加注重改善民生，让改革发展成果更多地惠及百姓

继续扩大就业。推进全民创业促就业工程，重点做好困难家庭、高校毕业生、零就业家庭和农民工就业，实名制培训下岗失业人员和进城务工农民2.6万人，安置实名制就业4万人。维护劳动者合法权益，把铁岭打造成全民创业型城市和不拖欠农民工工资的城市。完善社会保障体系。进一步巩固养老保险市级统筹，做好西丰县新型农村社会养老保险制度试点工作。城镇居民基本医疗保险覆盖面达到90%以上，不断提高新农合保障水平。完成农村低保边缘户D级危房改造2000户。加强城市弃管楼整治，完成农村改厕1.2万户，改善城乡居民居住环境。建设农村饮水安全工程100处，解决10万人饮水安全问题。

（八）全面繁荣各项社会事业，促进经济社会协调发展

深入开展教育年活动，重点实施教育园区建设、教育质量提升、职业教育发展等10大工程。扎实推进医药卫生体制改革，加快形成包括公共卫生、医疗服务、医疗保障、药品供应保障四位一体的基本医疗卫生制度。抓好甲型H1N1流感防控工作，确保不发生大的疫情。深化文化体制改革，抓好城乡文化设施建设，加快发展文化事业和文化产业。做好第六次人口普查工作，综合治理出生人口性别比偏高问题。以全民健身工程为载体，广泛开展群众性体育活动，做好辽宁省第十一届运动会的组织参赛工作。继续推进有线广播电视数字化暨文化信息资源共享工程。广泛开展文明单位、文明乡村、文明城市三大创建和城乡共建活动，加强以未成年人为重点的思想道德建设，提升全社会的文明素质。

（九）全力维护社会稳定，努力构建和谐铁岭

扎实推进平安铁岭建设。深入开展打黑除恶专项斗争，继续加强公安信息化建设，提高打击犯罪能力。进一步落实社会治安综合治理措施，全面推进“五五”普法进程，认真排查调处各种矛盾纠纷，最大限度地减少违法犯罪行为。建立健全安全生产责任体系，坚持不懈地抓好安全生产大检查和专项整治，杜绝重特大事故。探索建立食品信息化监管新模式，确保食品质量安全。严格落实信访责任制，充分发挥信访大厅作用，完善信访案件联动处理机制，千方百计为人民群众排忧解难。加快应急平台建设，进一步完善应急管理体制，妥善处置各类突发事件。增进民族团结，维护宗教稳定。

（十）切实加强政府自身建设，努力建设人民满意政府

加强民主政治建设。自觉接受人大依法监督和政协民主监督，坚持重大事项向人大报告、向政协通报制度，认真执行人大及其常委会的各项决议、决定，积极支持人民政协履行职能，认真办理人大代表建议和政协提案，加强与民主党派、工商联、无党派人士、人民团体和社会各界人士的联系，努力做到民主决策、科学决策。加强政府法制建设。全面落实依法行政责任制，进一步提高依法行政水平，深化行政执法体制改革，逐步实现执法重心下移。加强软环境建设。深入开展软环境建设年活动，加快转变政府职能，进一步减少审批事项，继续向县（市）区下放权力，全面推行行政权力公开透明运行。加快推广四级联动的公共行政服务新机制，努力建设服务型政府。加强机关作风建设。克服官僚主义、形式主义，开短会、讲短话、发短文，深入实际，深入基层，深入群众，认真开展调查研究，做到查实情、讲实话、办实事。加强政务督查，提高绩效考评的严肃性和权威性，狠抓工作落实。加强政府廉政建设。完善惩治和预防腐败体系，建立统一规范的公共资源交易中心，深入开展工程建设领域专项治理，强化监督制约机制，从源头上遏制腐败。严肃查处各种违法违纪案件，对典型案件公开曝光，努力建设廉洁政府。

在全力抓好今年各项工作的同时，全面总结“十一五”规划执行情况，认真编制好“十二五”规划。

各位代表，今天的铁岭已驶入科学发展的快车道，踏上奋力赶超的新征程。让我们更加紧密地团结在以胡锦涛同志为总书记的党中央周围，高举中国特色社会主义伟大旗帜，在中共铁岭市委的正确领导下，满怀发展激情，开创伟大事业，为夺取新铁岭大铁岭建设的新胜利而努力奋斗！
